# Remdesivir-Induced Bradycardia and Mortality in SARS-CoV-2 Infection, Potential Risk Factors Assessment: A Systematic Review and Meta-Analysis

**DOI:** 10.3390/jcm12247518

**Published:** 2023-12-05

**Authors:** Ming-Ying Ai, Wei-Lun Chang, Chia-Jui Yang

**Affiliations:** 1 Department of Pharmacy, Far Eastern Memorial Hospital, New Taipei City 22060, Taiwan; m120103008@tmu.edu.tw (M.-Y.A.); littlewind@pharmacist.tw (W.-L.C.); 2 Department of Internal Medicine, Far Eastern Memorial Hospital, New Taipei City 22060, Taiwan; 3 School of Medicine, National Yang Ming Chiao Tung University, Taipei 11217, Taiwan

**Keywords:** remdesivir, bradycardia, COVID-19, mortality, obesity, chronic kidney disease

## Abstract

**Background**: The efficacy of remdesivir in reducing disease severity among COVID-19-infected patients has been established, but concerns have emerged regarding the potential side effects of bradycardia. The aim of this study was to investigate the association between remdesivir-induced bradycardia and mortality, while also identifying the related risk factors. **Materials and methods**: The PubMed/Medline, Cochrane Central and ClinicalTrials.gov databases were searched. Randomized controlled trials and prospective or retrospective cohort studies were included (through 14 July 2023). The random-effects model was implemented using Comprehensive Meta-Analysis software version 3.0 to examine the outcomes. **Results**: A total of 12 prospective or retrospective studies involving 7674 patients were analyzed. The primary outcomes revealed a significant association between remdesivir administration and bradycardia development (Odds ratio = 2.556, 95% CI = 2.049–3.188, *p* < 0.001). However, no statistically significant increase in the mortality rate was observed among patients with bradycardia during remdesivir treatment (Odds ratio = 0.872, 95% CI = 0.483–1.576, *p* = 0.651). The secondary outcome demonstrated a significant association between chronic kidney disease (CKD) and remdesivir-induced bradycardia (OR: 1.251, 95% CI: 1.003–1.561, *p* = 0.047). Moreover, patients with obesity (OR = 1.347, 95% CI = 1.098–1.652, *p* = 0.004) were more likely to experience remdesivir-induced bradycardia. **Conclusions**: Although a higher risk of bradycardia occurred during remdesivir treatment, the occurrence of remdesivir-induced bradycardia did not lead to higher mortality. Our study also identified patients with obesity and CKD as high-risk subgroups for experiencing bradycardia during remdesivir treatment.

## 1. Introduction

Remdesivir (GS-441524), an antiviral medication, was designed for the treatment of a variety of viral infections, including coronavirus disease 2019 (COVID-19). When the COVID-19 pandemic began in late 2019, remdesivir quickly became a focal point of clinical trials [1]. Its potential to inhibit the replication of SARS-CoV-2 made it a promising candidate for treating COVID-19 patients. Numerous randomized controlled trials were conducted to assess the safety and efficacy of remdesivir in reducing disease severity and hospitalization duration [2,3]. However, concerns have been raised regarding its potential side effects, particularly its impact on cardiac function, including arrhythmia and bradycardia [4,5].

Bradycardia is described as an abnormally lower than normal heart rate (often fewer than 60 beats per minute) as a result of an underlying medical condition or disease. Some common causes include sinus node dysfunction, atrioventricular (AV) block, myocarditis, hypothyroidism, and certain neurological disorders [6]. Numerous drugs, including beta-blockers, calcium channel blockers, antiarrhythmics, and certain psychiatric medications, have the potential to induce bradycardia as a side effect [7]. Bradycardia can disrupt normal cardiac function and lead to symptoms such as dizziness, fatigue, and fainting.

In recent research, there has been considerable interest in investigating the association between remdesivir treatment and the occurrence of bradycardia [8]. Although remdesivir has shown efficacy in treating COVID-19, it is crucial to explore its potential cardiovascular effects, identify risk factors that may result in bradycardia, and gain a deeper understanding of the underlying mechanism.

## 2. Materials and Methods

### 2.1. General Guidelines

This meta-analysis followed the PRISMA guidelines for reporting [9]. This study was registered in INPLASY (registration number: 202360066) [10].

### 2.2. Database Search and Identified Manuscripts

Two authors (Ming-Ying Ai, Chia-Jui Yang) conducted independent searches in the PubMed, Medline, Cochrane Central, and ClinicalTrials.gov databases using the keywords “Remdesivir AND bradycardia OR bradyarrhythmia”. A comprehensive search was conducted in these databases, starting from their inception and extending to the search date of 14 July 2023.

Originally, the two authors jointly screened titles and abstracts to determine study relevance. Thorough searches were conducted in PubMed, Medline, and other databases to identify potentially eligible trials. In cases of disagreement between the two authors, a third reviewer and the research author were consulted. No language restrictions were applied to the search process.

### 2.3. Inclusion and Exclusion Criteria

The PICO (population, intervention, comparison, outcomes) settings of the current meta-analysis were as follows: P: Humans infected with COVID-19; I: remdesivir treatment; C: no remdesivir treatment; O: occurrence of bradycardia/bradyarrhythmia.

The inclusion criteria were as follows: (1) randomized controlled trials and prospective or retrospective cohort studies; (2) bradycardia after remdesivir treatment was investigated; and (3) participants were diagnosed with COVID-19 and received remdesivir treatment, and additional therapeutic agents were allowed.

The exclusion criteria were as follows: (1) case reports or case series; (2) participants were infected with Ebola or other viruses (without COVID-19 infection) and received remdesivir treatment; and (3) participants had COVID-19 and did not receive remdesivir treatment.

### 2.4. Methodological Quality Appraisal

The studies included in our meta-analysis consisted of both prospective and retrospective cohort studies. The quality assessment was conducted using the widely used Newcastle–Ottawa Scale (NOS), which assesses the quality and bias of cohort and case–control studies [11]. The NOS comprises three domains: selection of study groups, comparability of groups, and assessment of outcomes. Each domain is assigned a specific score, and the total score reflects the overall methodological quality.

### 2.5. Primary Outcomes

The main objective of this study was to evaluate the potential association between remdesivir administration and the occurrence of bradycardia. Our analysis encompassed retrospective and prospective cohort studies. The correlation between remdesivir-induced bradycardia and mortality was also investigated as the primary outcome. Sensitivity tests and analyses of publication bias were also conducted.

### 2.6. Secondary Outcomes

The secondary outcomes involved the evaluation of the risk factors associated with remdesivir-induced bradycardia. The potential risk factors assessed included sex, age, obesity, cardiovascular diseases (CVDs), hypertension, diabetes, thyroid disease, chronic kidney disease (CKD), intensive care unit (ICU) admission, and beta-blocker or antiarrhythmic drug usage. Sensitivity tests and analyses of publication bias were also conducted. For calculation, the value of zero was replaced with 0.5 for cells with zero events in our meta-analysis study [12].

### 2.7. Statistical Analysis

Heterogeneity was observed in the target populations across the included studies, and the random-effects model was implemented using Comprehensive Meta-Analysis software (version 3, Biostat, Englewood, NJ, USA) in this article [13]. A *p* ≤ 0.05 (two-tailed) was considered statistically significant. The Hedges’ g, odds ratios (ORs), and 95% confidence intervals (CIs) were used to qualify the study outcomes. The Hedges’ g values of 0.2, 0.5, and 0.8 represent small, moderate and large effect sizes [14]. To assess the degree of heterogeneity among studies, I^2^ and Cochran’s Q statistics were also determined. I^2^ values of 25%, 50%, and 75% were employed to indicate low, moderate, and high levels of heterogeneity, respectively.

To ensure the reliability of this meta-analysis, sensitivity analyses were conducted using the one study removed method. This method involved examining whether the summary effect size significantly changed after excluding a specific trial from the analysis [11]. The potential publication bias was assessed following the *Cochrane Handbook for Systematic Reviews of Interventions* [15]. Funnel plots were generated and visually examined to evaluate the publication bias.

We also performed the trial sequential analysis (TSA) to control for type I and type II errors for remdesivir-induced bradycardia-related mortality. A fixed-effects model was used to construct the cumulative Z curve. Sequential boundaries were calculated based on the O’Brien–Fleming spending function, with the significance levels set at 0.05 and a power of 95%. The OR and 95% confidence intervals (CIs) were estimated using the fixed-effects model through the Mantel–Haenszel method. For remdesivir-induced bradycardia, we selected a relative risk reduction of 10%. For mortality associated with bradycardia, we selected a relative risk reduction of 20%. The TSA version 0.9.5.5b (reviewed in November 2016) software was utilized to analyze the cumulative effect of the studies on mortality.

## 3. Results

### 3.1. Study Identification and Selection

**Figure 1:**
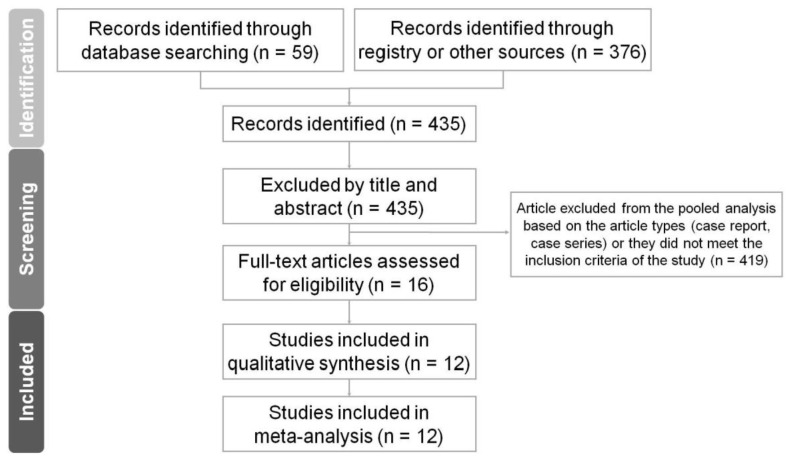
PRISMA 2020 flowchart for the current meta-analysis.

Figure 1, the PRISMA flowchart, illustrates the sequential steps taken to identify and select studies for the analysis. Initially, a thorough search of relevant databases was conducted using appropriate keywords and search terms. The Newcastle–Ottawa Scale (NOS) scores for the methodological quality assessment results are shown in Table 1. A total of ten retrospective and two prospective cohort studies were included in our meta-analysis (Table 2). 

Our meta-analysis was comprised of ten retrospective studies and two prospective studies, involving a total of 7674 adult individuals [16,17,18,19,20,21,22,23,24,25,26,27]. All included studies were conducted in adult populations. We conducted an analysis on the correlation between mortality and remdesivir-induced bradycardia. Additionally, we examined the risk factors associated with remdesivir-induced bradycardia.

### 3.2. Primary Outcome: The Association between Mortality and Remdesivir-Induced Bradycardia

**Figure 2:**
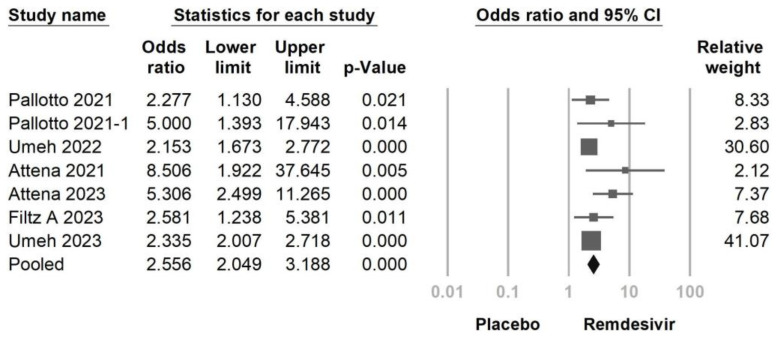
Forest plot of remdesivir-induced bradycardia or control [16,17,18,22,24,25,27].

**Figure 3:**
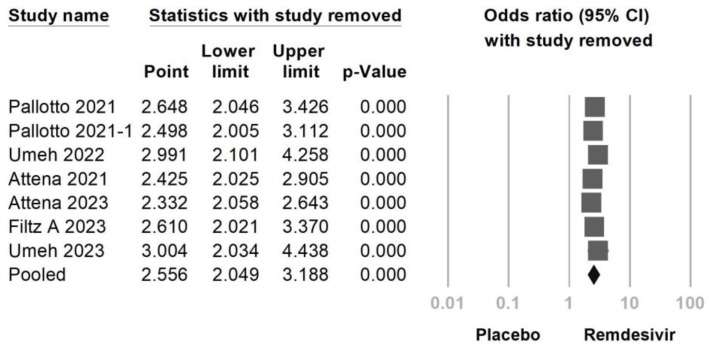
Sensitivity analysis of remdesivir-induced bradycardia or control utilizing the one study removed method [16,17,18,22,24,25,27].

**Figure 4:**
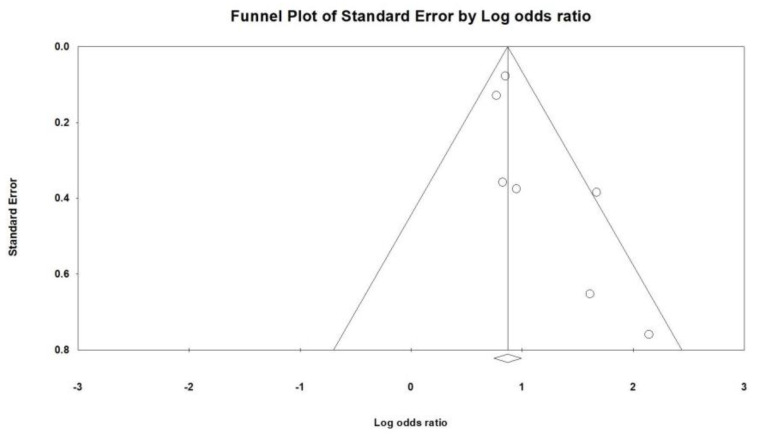
Funnel plot of remdesivir or control-related bradycardia.

The main objective of this meta-analysis was to evaluate the incidence of bradycardia, both with and without the use of remdesivir, and its association with mortality in cases of remdesivir-induced bradycardia. Combining data from seven studies (Figure 2), the results indicated that the use of remdesivir was associated with a higher likelihood of bradycardia compared to the control group (OR = 2.556, 95% CI: 2.049–3.188, *p* < 0.001, I^2^ = 34.73%). However, a mild to moderate heterogeneity was observed. A sensitivity analysis was conducted by excluding one study. Even when one of the included studies was removed, the overall effect sizes remained statistically significant (Figure 3). The funnel plot of the seven studies did not display asymmetry in the distribution of effect sizes, indicating no potential publication bias, as suggested by Egger’s regression (*p* = 0.057) (Figure 4).

**Figure 5:**
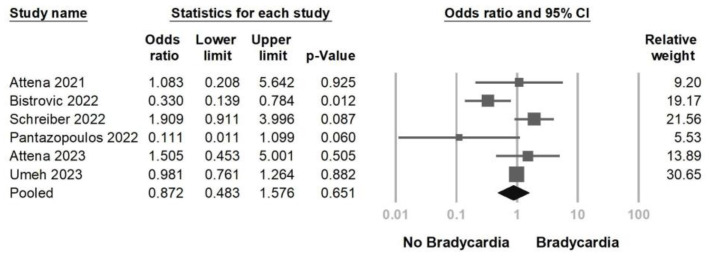
Forest plot of remdesivir-induced bradycardia associated with mortality [16,19,20,21,24,27].

**Figure 6:**
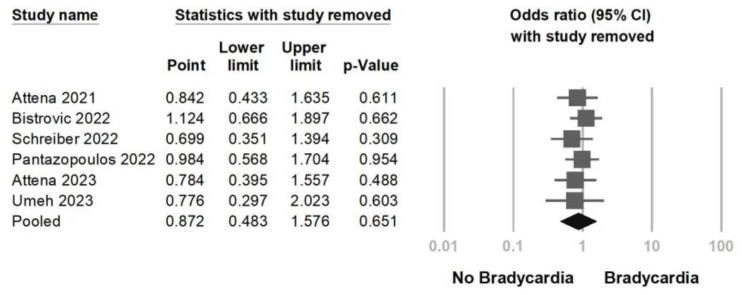
Sensitivity analysis of remdesivir-induced bradycardia associated with mortality utilizing the one study removed method [16,19,20,21,24,27].

Next, we aimed to investigate whether remdesivir-induced bradycardia was associated with a higher mortality than that in the non-remdesivir-induced bradycardia group. Six studies were included in this analysis (Figure 5), and the results indicated no significant difference between the two groups (OR = 0.872, 95% CI = 0.483–1.576, *p* = 0.651, I^2^ = 62.0%). A mild to moderate heterogeneity was observed. The sensitivity analysis, which involved the removal of one study, yielded consistent results (Figure 6). Egger’s regression did not indicate potential publication bias (*p* = 0.621) (Figure S1).

### 3.3. Secondary Outcomes: Risk Factor of Remdesivir-Induced Bradycardia

The secondary outcomes were to evaluate the risk factors associated with remdesivir-induced bradycardia. We analyzed the occurrence of bradycardia based on the patient characteristics, including sex, age, obesity, cardiovascular diseases (CVDs), hypertension, diabetes, thyroid disease, chronic kidney disease (CKD), intensive care unit (ICU) admission, and beta-blocker or antiarrhythmic drug usage.

**Figure 7:**
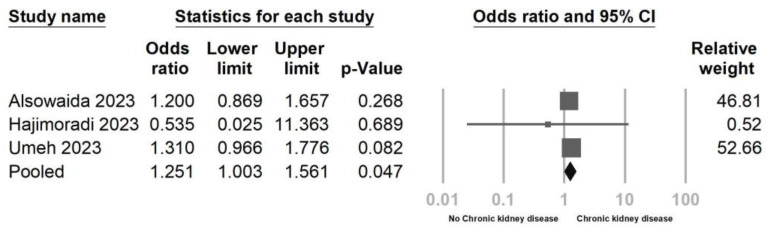
Forest plot of remdesivir-induced bradycardia associated with chronic kidney disease (CKD) [23,26,27].

**Figure 8:**
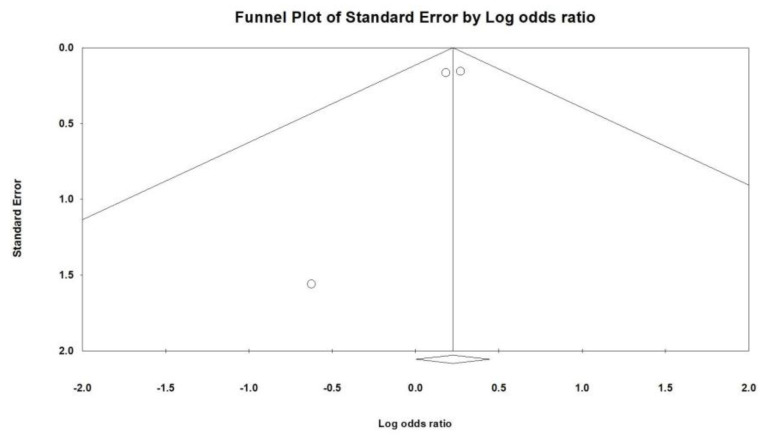
Funnel plot of remdesivir−induced bradycardia associated with chronic kidney disease (CKD).

**Figure 9:**
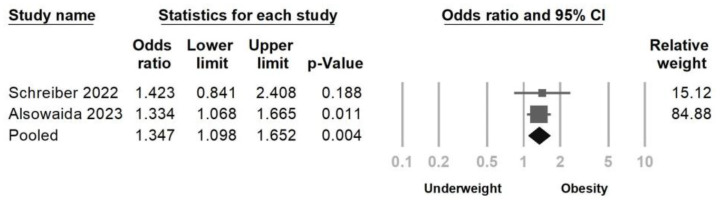
Forest plot of remdesivir-induced bradycardia associated with obesity [21,23].

Three studies were included to evaluate the impact of CKD, as shown in Figure 7. The findings revealed a significant association between CKD and remdesivir-induced bradycardia (OR = 1.251, 95% CI = 1.003–1.561, *p* = 0.047, I^2^ = 0.0%), with no observed heterogeneity. Additionally, Egger’s regression test (Figure 8) demonstrated no indication of publication bias (*p* = 0.364). Two studies were incorporated to assess the effect of obesity. The results indicated that patients with obesity were more likely to experience remdesivir-induced bradycardia (OR = 1.347, 95% CI = 1.098–1.652, *p* = 0.004, I^2^ = 0.0%, Figure 9).

Regarding the assessment of sex (OR = 0.998, 95% CI = 0.811–1.228, *p* = 0.982, I^2^ = 38.04%, Egger’s *p* = 0.380), age (OR = 0.286, 95% CI = −0.154–0.725, *p* = 0.202, I^2^ = 94.16%, Egger’s *p* = 0.157), CVDs (OR = 0.977, 95% CI = 0.751–1.271, *p* = 0.861, I^2^ = 30.66%, Egger’s *p* = 0.932), hypertension (OR = 1.253, 95% CI = 0.935–1.679, *p* = 0.131, I^2^ = 32.29%, Egger’s *p* = 0.133), diabetes (OR = 0.928, 95% CI = 0.770–1.118, *p* = 0.430, I^2^ = 0.0%, Egger’s *p* = 0.555), thyroid disease (OR = 1.344, 95% CI = 0.984–1.835, *p* = 0.063, I^2^ = 0.0%, Egger’s *p* = 0.474), ICU admission (OR = 1.080, 95% CI = 0.612–1.905, *p* = 0.791, I^2^ = 83.66%, Egger’s *p* = 0.429), and beta-blocker (OR = 1.038, 95% CI = 0.779–1.382, *p* = 0.801, I^2^ = 0.0%, Egger’s *p* = 0.973) or antiarrhythmic drug usage (OR = 0.814, 95% CI = 0.125–5.288, *p* = 0.829, I^2^ = 70.68%, Egger’s *p* = 0.573), all results indicated no significant difference between the remdesivir-induced bradycardia and bradycardia-free groups (Figures S2–S10). The funnel plots showed no evidence of publication bias (Figures S11–S19).

### 3.4. Trial Sequential Analysis (TSA) of Remdesivir-Induced Bradycardia and Remdesivir-Induced Bradycardia-Related Mortality

To control the type I and type II errors in the context of remdesivir-induced bradycardia and its related mortality, we utilized the trial sequential analysis (TSA). The analysis included a total of 2101 patients to determine the superiority or neutrality boundary concerning remdesivir-induced bradycardia. The Z curve indicated that bradycardia occurred more frequently with remdesivir treatment. Notably, this evidence indicated that the number of enrolled patients was almost sufficient to draw a conclusion (Figure S20).

Regarding remdesivir-induced bradycardia-related mortality, the TSA included 2438 patients to establish the superiority or neutrality boundary. The Z curve showed parallelism with the bradycardia group’s superiority zone, indicating that remdesivir-induced bradycardia did not result in a higher mortality rate than non-remdesivir-induced bradycardia. However, the curve remained within the futility area for all trials, suggesting that approximately 500 more patients were needed to reach a conclusive result (Figure S21).

## 4. Discussion

Based on the results obtained from our meta-analysis, we found a significant association between the use of remdesivir and the occurrence of bradycardia. However, there was no significant difference in the mortality rates between patients who experienced remdesivir-induced bradycardia and those who did not. These findings indicate that while remdesivir may increase the risk of bradycardia, it may not necessarily lead to higher mortality among patients with COVID-19. The incidence of remdesivir-induced bradycardia ranged from 16.8% to 73.8%. Given this wide range, it is important to identify the risk factors associated with remdesivir-induced bradycardia.

Regarding the secondary outcomes, our analysis revealed that patients with CKD and obesity might be at a higher risk of developing bradycardia when treated with remdesivir. However, other factors, such as age, sex, CVDs, hypertension, diabetes, thyroid disease, ICU admission, beta-blocker usage, and antiarrhythmic drug usage, were not found to be significantly associated with remdesivir-induced bradycardia. Notably, our study is the first meta-analysis to investigate the risk factors associated with remdesivir-induced bradycardia.

Several case reports and studies have also reported cases of bradycardia among patients receiving remdesivir therapy [28]. However, the exact mechanisms underlying this association remain unclear. One possible mechanism is direct cardiac toxicity caused by the active form of remdesivir, which requires intracellular metabolism. The active form interacts with cellular components, including ion-channel function, leading to altered electrical signaling within the heart and subsequently resulting in bradycardia [29,30]. Another study proposed a hypothesis explaining the bioaccumulation of the intermediate metabolite GS-441524 of remdesivir, which leads to the exogenous activation of cardiac adenosine A1 receptors. This activation is attributed to the structural resemblance between adenosine and GS-441524. The extended half-life of GS-441524 can contribute to the continuous activation of adenosine A1 receptors and cause cardiac conduction abnormalities [31].

However, it is important to consider that SARS-CoV-2 itself can also lead to cardiovascular complications, including bradycardia, through autonomic nervous system dysfunction and impairment of normal sinus node activity. Kumar et al. conducted a study involving 28.7% of COVID-19 patients who received remdesivir treatment in order to investigate the relationship between bradycardia and mortality. The findings suggested that the development of bradycardia was associated with a higher mortality rate [32]. However, the specific impact of remdesivir-induced bradycardia on the mortality rate remained unclear. In our meta-analysis, we found no significant association between remdesivir-induced bradycardia and a higher mortality rate.

Moreover, the consideration of drug-drug interactions is also important. In the treatment of COVID-19, remdesivir is frequently coadministered with other medications known to have bradycardia effects, such as beta-blockers or calcium channel blockers. A previous study suggested that coadministration of these drugs, which interfere with the heart rate, may increase the risk of remdesivir-induced bradycardia [33]. However, our meta-analysis, which included three studies, found no evidence of an increased occurrence of bradycardia when remdesivir was coadministered with beta-blockers or antiarrhythmic drugs.

In our study, CKD was identified as one of the risk factors of remdesivir-induced bradycardia. The precise mechanism underlying remdesivir-induced bradycardia in CKD patients is not yet fully understood and requires further investigation. Nevertheless, several potential mechanisms have been proposed. One potential mechanism involves the accumulation of remdesivir metabolites in CKD patients, which have a prolonged half-life and may lead to sustained activation of adenosine A1 receptors. Although remdesivir does not accumulate in the body, its metabolite GS-441524 accumulates approximately twofold after multiple once-daily doses and should be mainly excreted by the kidneys [34].

Adenosine interacts with four receptor subtypes located on the surfaces of various cells throughout the body: A1, A2, and A3. Activation of A1 receptors induces negative chronotropic and dromotropic effects [35]. Within the heart, adenosine primarily binds to A1 receptors located in the atria. This binding results in reduced cyclic adenosine monophosphate (cAMP) production as well as the inhibition of protein kinase A, blocking of calcium channels, and opening of potassium-gated channels. The suppression of calcium influx and the enhancement of potassium play a crucial role in inhibiting atrioventricular (AV) node conduction, leading to a shortened action potential duration and increased refractoriness [36]. The molecular modeling indicates a significantly high level of binding affinity for GS-441524 as an exogenous ligand to the adenosine A1 receptor. The bioaccumulation of GS-441524 is hypothesized to lead to an exogenous activation of cardiac adenosine A1 receptors [31]. Stimulation of these receptors is recognized to induce a myocardial depressant effect by slowing conduction and suppressing cardiac pacemaker function.

Furthermore, CKD patients often have multiple comorbidities, including electrolyte imbalances and cardiovascular diseases, which can further contribute to the development of bradycardia. Currently, most international guidelines and US FDA fact sheets for the treatment of COVID-19 do not provide specific recommendations regarding dose adjustments for those with renal insufficiency [37,38,39]. The pharmacokinetics and mechanisms involved should be further investigated in these populations to provide proper dosage adjustment recommendations.

Another identified risk factor in our study was obesity. We observed an increased risk of remdesivir-induced bradycardia among patients with obesity. There are several proposed explanations for this finding. Obesity can influence the metabolism and distribution of medications within the body. In individuals with obesity, changes in drug absorption, distribution, metabolism, and elimination may occur [40]. A previous study also showed that the factors influencing GS-441524 (remdesivir metabolite) serum concentration included a lower estimated glomerular filtration rate (eGFR) and a body mass index (BMI) of ≥25 kg/m^2^. These pharmacokinetic studies also demonstrated that obesity might contribute to expanding the volume of distribution (Vd) of GS-441524 [41]. Such alterations can result in prolonged GS-441524 effects, potentially increasing the risk of side effects such as bradycardia. Additionally, obesity is associated with structural changes in the heart, including left ventricular hypertrophy and increased adipose tissue around the heart, which can affect cardiac function and electrical conduction, making the heart more susceptible to arrhythmias and bradycardia. Dysregulation of the autonomic nervous system, which controls the heart rate and rhythm, is another proposed mechanism. Imbalances in sympathetic and parasympathetic activities can disrupt the normal heart rate control mechanisms, potentially leading to bradycardia in response to remdesivir.

According to previous guidelines, the standard remdesivir treatment course consists of five days, with a loading dose of 200 mg on the first day followed by a maintenance dose of 100 mg for the subsequent four days. The treatment course could be extended to a maximum of 10 days [38,39]. In our review, most of the included studies followed the standard treatment duration, except for the study conducted by Alsowaida et al., which extended the treatment to 10 days. However, despite the extended treatment duration, we did not observe a significantly higher incidence of bradycardia compared to the other studies. The potential impact of different treatment regimens on the incidence of remdesivir-induced bradycardia should be investigated further.

Our study, previous case reports, and studies [42] have all emphasized the importance of evaluating patient characteristics associated with bradycardia during remdesivir treatment. Gaining insights into the risk factors associated with remdesivir-induced bradycardia is crucial for making informed decisions regarding patient management and treatment approaches. However, the available data on the incidence, severity, and clinical implications of remdesivir-induced bradycardia are limited. It is crucial to undertake comprehensive large-scale randomized controlled trials and pharmacovigilance surveillance to evaluate the frequency, risk factors, and clinical significance of remdesivir-induced bradycardia. A better understanding of the potential cardiac effects of remdesivir and the establishment of appropriate monitoring protocols can offer essential guidance to clinicians in optimizing patient safety and effectively managing any cardiovascular complications that may arise from the treatment.

## 5. Conclusions

Based on our meta-analysis findings, a higher risk of bradycardia was found in the remdesivir group compared to the control group. However, it is noteworthy that remdesivir-induced bradycardia did not lead to a higher mortality rate. Our study identified patients with obesity and CKD as high-risk subgroups for developing bradycardia during remdesivir treatment. Health care providers should take these risk factors into account when prescribing remdesivir and closely monitor patients for any signs or symptoms of bradycardia. Prompt interventions should be initiated if bradycardia occurs during the remdesivir treatment. Further large-scale randomized controlled trials are necessary to investigate the underlying mechanisms and validate the association between these risk factors and remdesivir-induced bradycardia.

## Figures and Tables

**Table 1:** Detailed quality assessment of the included studies using the Newcastle-Ottawa Scale (NOS).

First Author	Year	S1 ^1^	S2	S3	S4	C1 ^2^	E1 ^3^	E2	E3	Total
**Attena, E.** [16]	**2021**	*	*	*		**	*	*	*	8
**Pallotto, C.** [17]	**2021**	*	*	*		**	*	*	*	8
**Pallotto, C.** [18]	**2021**	*	*	*	*	**	*	*	*	9
**Bistrovic, P.** [19]	**2022**	*		*	*	*	*	*	*	7
**Pantazopoulos** [20]	**2022**	*	*	*	*	*	*	*	*	8
**Schreiber** [21]	**2022**	*	*	*	*	*	*	*	*	8
**Umeh, C.** [22]	**2022**	*	*	*	*	**	*	*	*	9
**Alsowaida, Y.S.** [23]	**2023**	*		*	*	*	*	*	*	7
**Attena, E.** [24].	**2023**	*	*	*	*	**	*	*	*	9
**Filtz, A.** [25]	**2023**	*	*	*	*	**	*	*	*	9
**Hajimoradi, M.** [26]	**2023**	*		*	*	*	*	*	*	7
**Umeh, C.** [27]	**2023**	*	*	*	*	**	*	*	*	9

^1^ S: The Selection part assessment. ^2^ C: The Comparability part assessment. ^3^ E: The Outcome part assessment. *: One point of the Newcastle-Ottawa scale, studies get one point at each category once they met the criteria. **: Two points of the Newcastle-Ottawa scale in the comparability part assessment.

**Table 2:** Summary of the studies investigating the effect of remdesivir-induced bradycardia in the enrolled participants.

First Author and Year	Country	Population	Participants	Dosage	Duration	Incidence ^1^	Bradycardia Definition (Beat/min)	Study Design	Additional Therapies	Quality Assessment	Funding/Grants/ Support
Attena, E. 2021 [16]	Italy	Hospitalized adult patients	RDV: 100 Control: 66	LD: 200 mg MD: 100 mg	5–10 days were allowed	21/100 (21%)	HR < 50	Prospective Cohort study	Azithromycin, Dexamethasone, Heparin	8	N/A
Pallotto, C. 2021 [17]	Italy	Hospitalized adult patients	RDV: 62 Control: 79	LD: 200 mg MD: 100 mg	5 days	29/62 (46.8%)	^2^ HR < 60 × 2 or HR < 50 × 1	Retrospective Cohort Study	Steroids, LMWH	8	N/A
Pallotto, C. 2021 [18]	Italy	Hospitalized adult patients	RDV: 20 Control: 26	LD: 200 mg MD: 100 mg	5 days	12/20 (60.0%)	Compared pre and post △HR	Retrospective Cohort Study	Dexamethasone, LMWH	9	N/A
Bistrovic, P. 2022 [19]	Croatia	Hospitalized adult patients	RDV: 473	LD: 200 mg MD: 100 mg	5 days: 455/473 (96.2%) >5 days: 18/473 (3.8%)	79/473 (16.8%)	HR < 60	Retrospective Cohort Study	Steroids, LMWH	7	N/A
Pantazopoulos 2022 [20]	Greece	Hospitalized adult patients	RDV: 160	LD: 200 mg MD: 100 mg	5 days	118/160 (73.8%)	HR < 60	Retrospective Cohort Study	Dexamethasone, LMWH	8	N/A
Schreiber 2022 [21]	USA	Hospitalized adult patients	RDV: 375	LD: 200 mg MD: 100 mg	5 days	182/375 (48.5%)	HR < 60	Retrospective Cohort Study	Not mention	8	N/A
Umeh, C. 2022 [22]	USA	Hospitalized adult patients	RDV: 507 Control: 609	LD: 200 mg MD: 100 mg	5 days	218/507 (43.0%)	HR < 60	Retrospective Cohort Study	Dexamethasone, Methylprednisolone	9	N/A
Alsowaida, Y.S. 2023 [23]	USA	Hospitalized adult patients	RDV: 1635	LD: 200 mg MD: 100 mg	10 days	606/1635 (37.1%)	HR < 60	Retrospective Cohort Study	Dexamethasone	7	N/A
Attena, E. 2023 [24]	Italy	Hospitalized adult patients	RDV: 200 Control: 200	LD: 200 mg MD: 100 mg	5 days	40/200 (20.0%)	HR < 50	Retrospective Cohort Study	Azithromycin, Dexamethasone	9	Università degli Studi della Campania Luigi Vanvitelli within the CRUI-CARE Agreement
Filtz, A. 2023 [25]	Italy	Hospitalized adult patients	RDV: 71 Control: 54	LD: 200 mg MD: 100 mg	5 days	40/71 (56.0%)	HR < 60	Retrospective Cohort Study	Any other additional therapeutic according to guideline was allowed	9	Italian Ministry of Health
Hajimoradi, M. 2023 [26]	Iran	Outpatients/ Hospitalized adult patients	RDV: 177	LD: 200 mg MD: 100 mg	5 days	48/177 (27.3%)	HR < 60	Prospective Cohort study	Tocilizumab, Dexamethasone	7	N/A
Umeh, C. 2023 [27]	USA	Hospitalized adult patients	RDV: 1493 Control: 1367	LD: 200 mg MD: 100 mg	5 days	801/1254 (63.9%)	^3^ HR < 60 × 2	Retrospective Cohort Study	Dexamethasone	9	N/A

RDV, Remdesivir; USA, United States of America; LD, Loading dose; MD, Maintaining dose; HR, Heart rate; LMWH, Low-molecular-weight heparin. ^1^ Age is presented as means ± standard deviations or as medians (ranges). ^2^ HR < 60 for two consecutive measurements. ^3^ HR < 60 on two separate occasions, a minimum of 4 h apart; N/A, Not applicable.

## Data Availability

All data are available, either analyzed as figures and tables presented in the current manuscript or as raw data, upon request by any external collaborator or reviewer.

## Supplementary Materials

The following supporting information can be downloaded at: https://www.mdpi.com/article/10.3390/jcm12247518/s1, Figure S1: Funnel plot of remdesivir-induced bradycardia associated with mortality; Figure S2: Forest plot of remdesivir-induced bradycardia associated with sex; Figure S3: Forest plot of remdesivir-induced bradycardia associated with age; Figure S4: Forest plot of remdesivir-induced bradycardia associated with cardiovascular diseases (CVDs); Figure S5: Forest plot of remdesivir-induced bradycardia associated with hypertension; Figure S6: Forest plot of remdesivir-induced bradycardia associated with diabetes; Figure S7: Forest plot of remdesivir-induced bradycardia associated with thyroid diseases; Figure S8: Forest plot of remdesivir-induced bradycardia associated with ICU admission; Figure S9: Forest plot of remdesivir-induced bradycardia associated with beta-blocker usage; Figure S10: Forest plot of remdesivir-induced bradycardia associated with antiarrhythmic drug usage; Figure S11: Funnel plot of remdesivir-induced bradycardia associated with sex; Figure S12: Funnel plot of remdesivir-induced bradycardia associated with age; Figure S13: Funnel plot of remdesivir-induced bradycardia associated with cardiovascular diseases (CVDs); Figure S14: Funnel plot of remdesivir-induced bradycardia associated with hypertension; Figure S15: Funnel plot of remdesivir-induced bradycardia associated with diabetes; Figure S16: Funnel plot of remdesivir-induced bradycardia associated with thyroid diseases; Figure S17: Funnel plot of remdesivir-induced bradycardia associated with ICU admission; Figure S18: Funnel plot of remdesivir-induced bradycardia associated with beta-blocker usage; Figure S19: Funnel plot of remdesivir-induced bradycardia associated with antiarrhythmic drug usage; Figure S20: Trial sequential analysis of the low risk of bias in studies comparing the impact on remdesivir-induced bradycardia; Figure S21: Trial sequential analysis of the low risk of bias in studies comparing the impact on remdesivir-induced bradycardia-related mortality.
