# Supplementary material for: Remdesivir-Induced Bradycardia and Mortality in SARS-CoV-2 Infection, Potential Risk Factors Assessment: A Systematic Review and Meta-Analysis
Source: J Clin Med. 2023 Dec 5;12(24):7518. doi: 10.3390/jcm12247518 (PMC10743390; doi:10.3390/jcm12247518)
Supplement: Supplementary file 1 [file jcm-12-07518-s001.zip › jcm-2672544-supplementary.pdf]

Supplementary materials

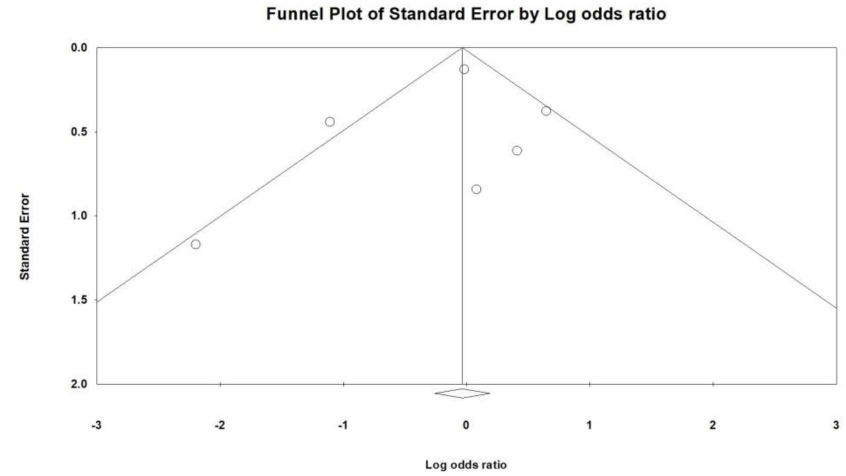

Figure S1. Funnel plot of remdesivir-induced bradycardia associated with mortality

Study name	Odds ratio	Lower limit	Upper limit	p-Value	Relative weight
Attena 2021	3.550	1.073	11.740	0.038	2.84
Pantazopoulos 2022	0.818	0.420	1.593	0.555	8.13
Schreiber 2022	0.940	0.620	1.425	0.771	16.61
Alsowaida 2023	1.078	0.882	1.318	0.461	34.01
Hajimoradi 2023	1.265	0.644	2.484	0.495	7.95
Umeh 2023	0.831	0.658	1.051	0.123	30.46
Pooled	0.998	0.811	1.228	0.982	

Statistics for each study

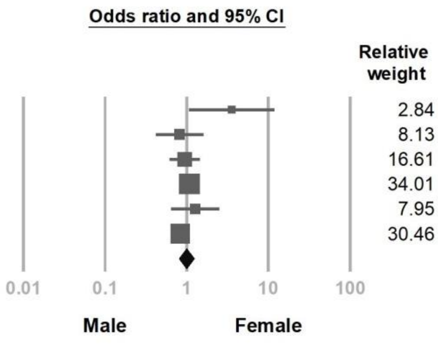

Figure S2. Forest plot of remdesivir-induced bradycardia associated with sex

Study name	Hedges's g	Lower limit	Upper limit	p-Value
Attena 2021	0.474	-0.008	0.956	0.054
Pantazopoulos 2022	0.048	-0.266	0.362	0.764
Schreiber 2022	0.016	-0.186	0.218	0.878
Alsowaida 2023	0.808	0.704	0.912	0.000
Hajimoradi 2023	0.068	-0.263	0.398	0.689
Pooled	0.286	-0.154	0.725	0.202

Statistics for each study

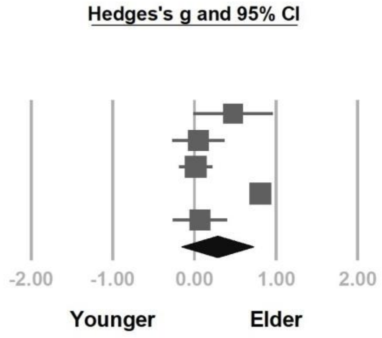

Figure S3. Forest plot of remdesivir-induced bradycardia associated with age

Study name	Odds ratio	Lower limit	Upper limit	p-Value	Relative weight
Pantazopoulos 2022	1.195	0.636	2.248	0.580	13.87
Schreiber 2022	1.063	0.365	3.092	0.911	5.59
Alsowaida 2023	1.125	0.895	1.415	0.314	45.16
Hajimoradi 2023	1.342	0.331	5.448	0.681	3.36
Umeh 2023	0.699	0.497	0.982	0.039	32.03
Pooled	0.977	0.751	1.271	0.861	

(Statistics for each study)

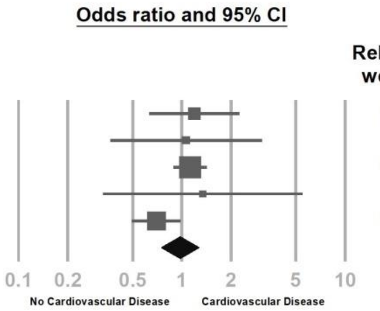

Figure S4. Forest plot of remdesivir-induced bradycardia associated with cardiovascular diseases (CVDs)

Study name	Odds ratio	Lower limit	Upper limit	p-Value	Relative weight
Attena 2021	1.061	0.401	2.802	0.906	8.09
Schreiber 2022	1.061	0.704	1.597	0.778	30.44
Alsowaida 2023	1.524	1.236	1.878	0.000	54.42
Hajimoradi 2023	0.687	0.241	1.960	0.483	7.05
Pooled	1.253	0.935	1.679	0.131	

(Statistics for each study)

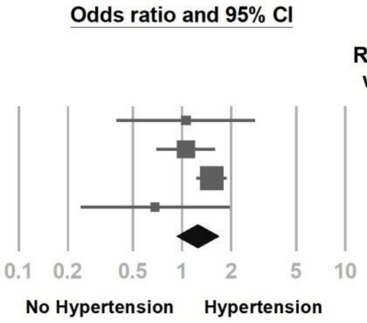

Figure S5. Forest plot of remdesivir-induced bradycardia associated with hypertension

Study name	Odds ratio	Lower limit	Upper limit	p-Value	Relative weight
Attena 2021	0.774	0.200	2.990	0.710	1.91
Schreiber 2022	0.999	0.665	1.501	0.996	21.03
Alsowaida 2023	0.899	0.723	1.118	0.338	73.29
Hajimoradi 2023	1.234	0.472	3.224	0.668	3.78
Pooled	0.928	0.770	1.118	0.430	

(Statistics for each study)

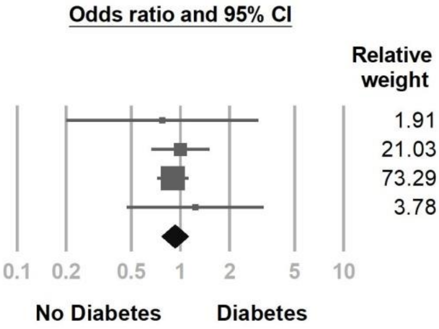

Figure S6. Forest plot of remdesivir-induced bradycardia associated with diabetes

Study name	Odds ratio	Lower limit	Upper limit	p-Value	Relative weight
Schreiber 2022	1.454	0.668	3.164	0.346	16.09
Alsowaida 2023	1.346	0.955	1.896	0.089	82.82
Hajimoradi 2023	0.370	0.019	7.306	0.514	1.09
Pooled	1.344	0.984	1.835	0.063	

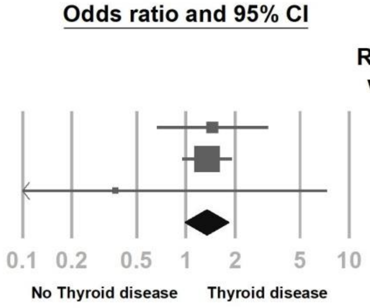

Figure S7. Forest plot of remdesivir-induced bradycardia associated with thyroid diseases

Study name	Odds ratio	Lower limit	Upper limit	p-Value	Relative weight
Attena 2021	0.934	0.183	4.769	0.935	9.14
Bistrovic 2022	0.430	0.202	0.917	0.029	22.30
Alsowaida 2023	1.943	1.508	2.503	0.000	34.44
Umeh 2023	1.133	0.865	1.484	0.366	34.12
Pooled	1.080	0.612	1.905	0.791	

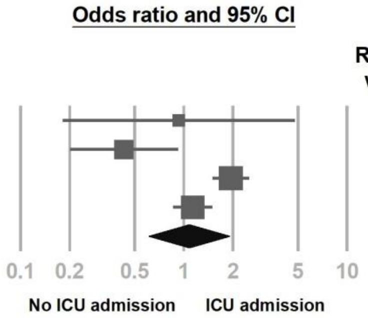

Figure S8. Forest plot of remdesivir-induced bradycardia associated with ICU admission

Study name	Odds ratio	Lower limit	Upper limit	p-Value	Relative weight
Attena 2021	0.602	0.199	1.820	0.368	6.71
Alsowaida 2023	1.030	0.753	1.408	0.853	84.00
Hajimoradi 2023	1.643	0.642	4.207	0.301	9.29
Pooled	1.038	0.779	1.382	0.801	

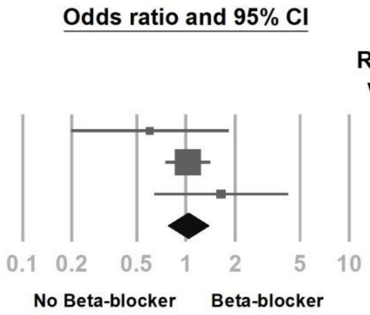

Figure S9. Forest plot of remdesivir-induced bradycardia associated with beta-blocker usage

Study name	Odds ratio	Lower limit	Upper limit	p-Value	Relative weight
Attena 2021	0.279	0.034	2.280	0.234	29.82
Pantazopoulos 2022	0.358	0.136	0.945	0.038	42.34
Hajimoradi 2023	8.927	0.903	88.210	0.061	27.84
Pooled	0.814	0.125	5.288	0.829	

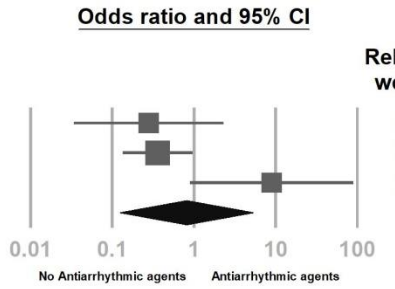

Figure S10. Forest plot of remdesivir-induced bradycardia associated with antiarrhythmic drug usage

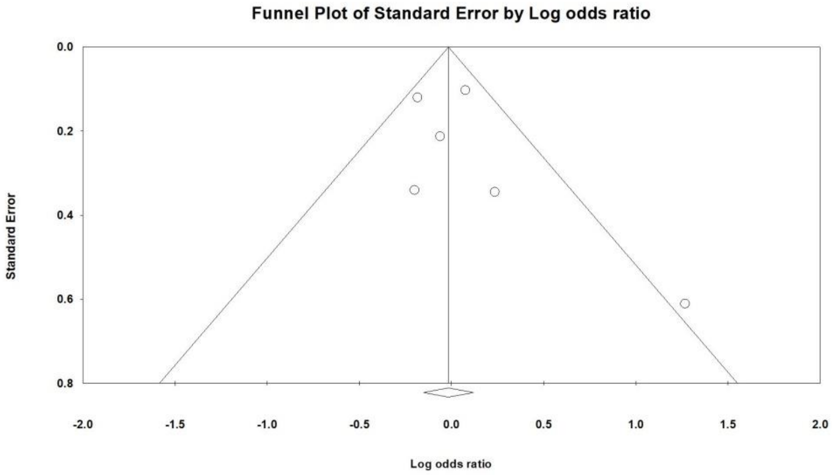

Figure S11. Funnel plot of remdesivir-induced bradycardia associated with sex

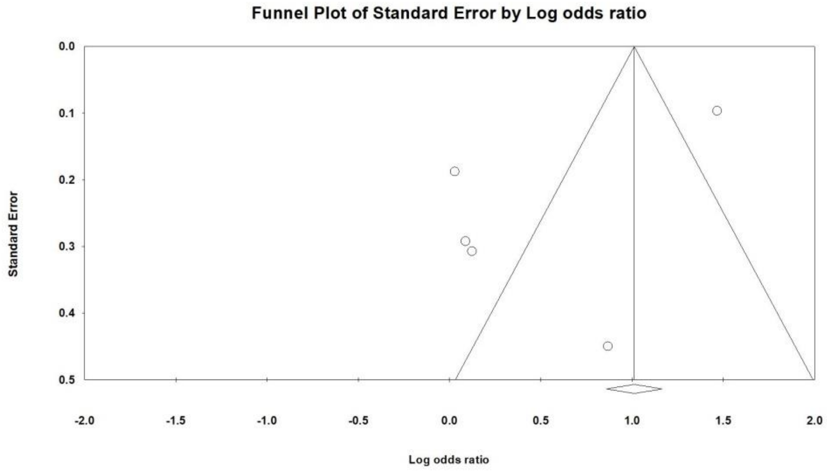

Figure S12. Funnel plot of remdesivir-induced bradycardia associated with age

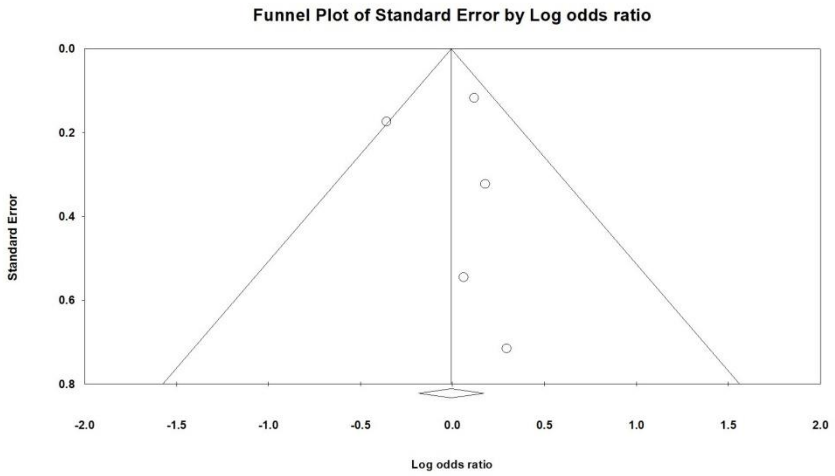

Figure S13. Funnel plot of remdesivir-induced bradycardia associated with cardiovascular diseases (CVDs)

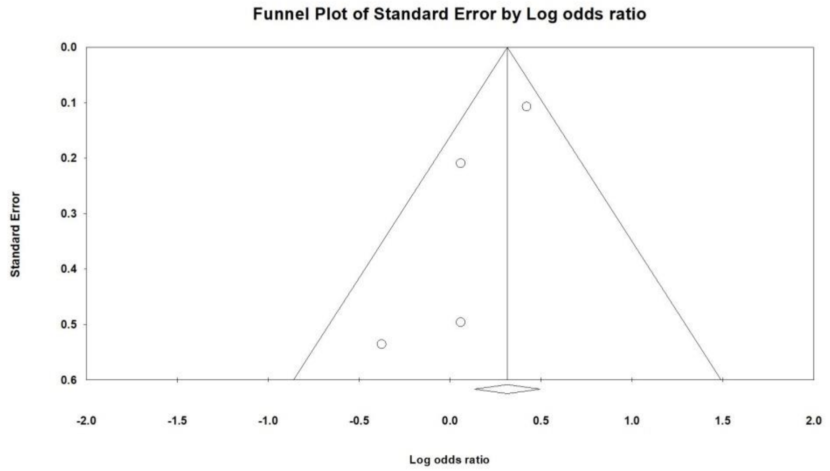

Figure S14. Funnel plot of remdesivir-induced bradycardia associated with hypertension

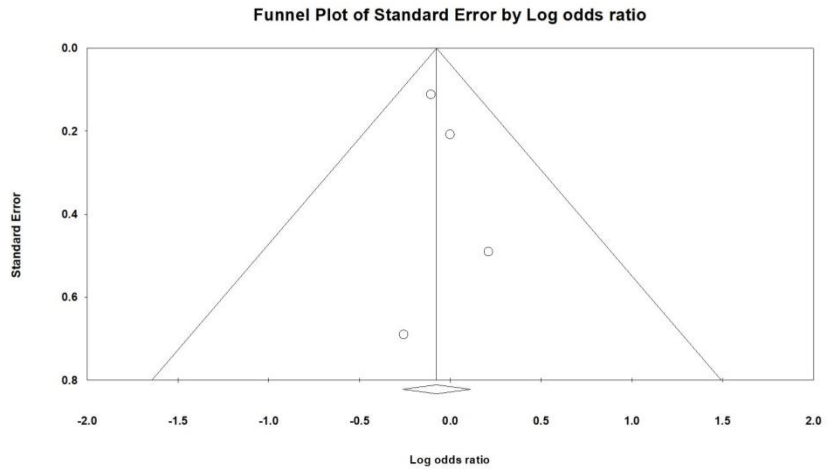

Figure S15. Funnel plot of remdesivir-induced bradycardia associated with diabetes

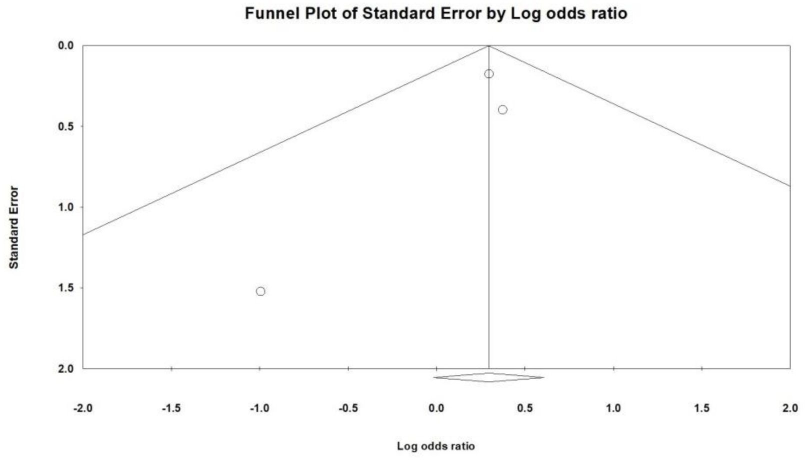

Figure S16. Funnel plot of remdesivir-induced bradycardia associated with thyroid diseases

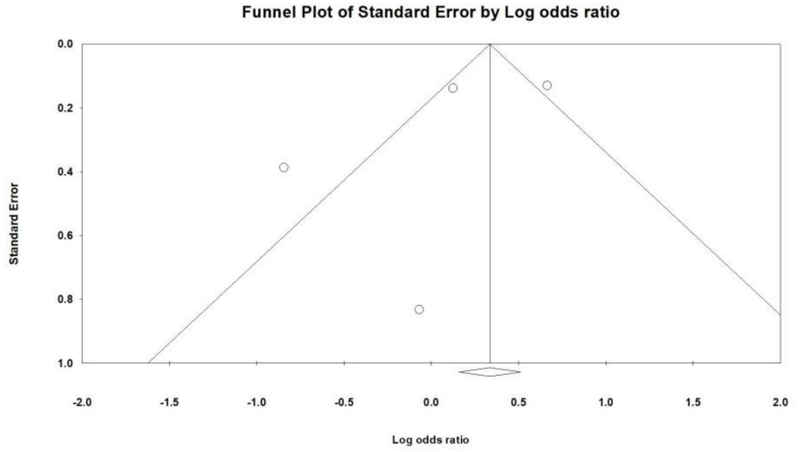

Figure S17. Funnel plot of remdesivir-induced bradycardia associated with ICU admission

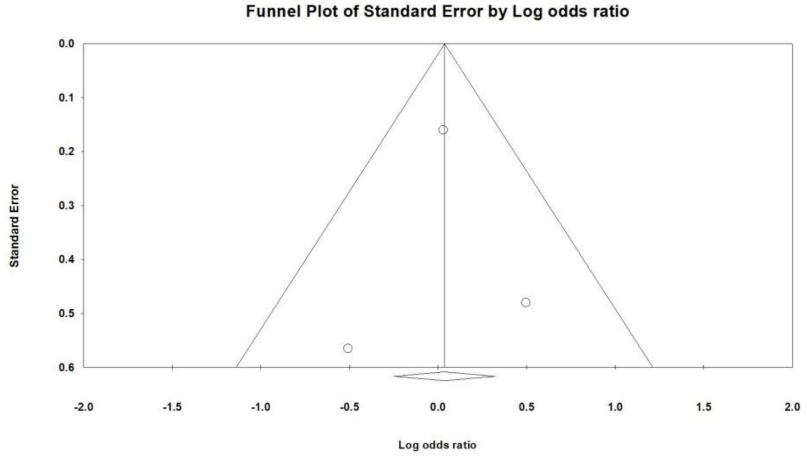

Figure S18. Funnel plot of remdesivir-induced bradycardia associated with beta-blocker usage

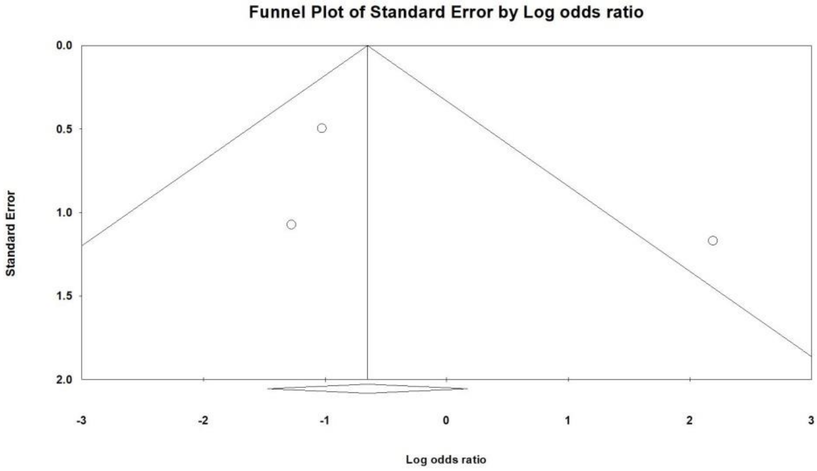

Figure S19. Funnel plot of remdesivir-induced bradycardia associated with antiarrhythmic drug usage

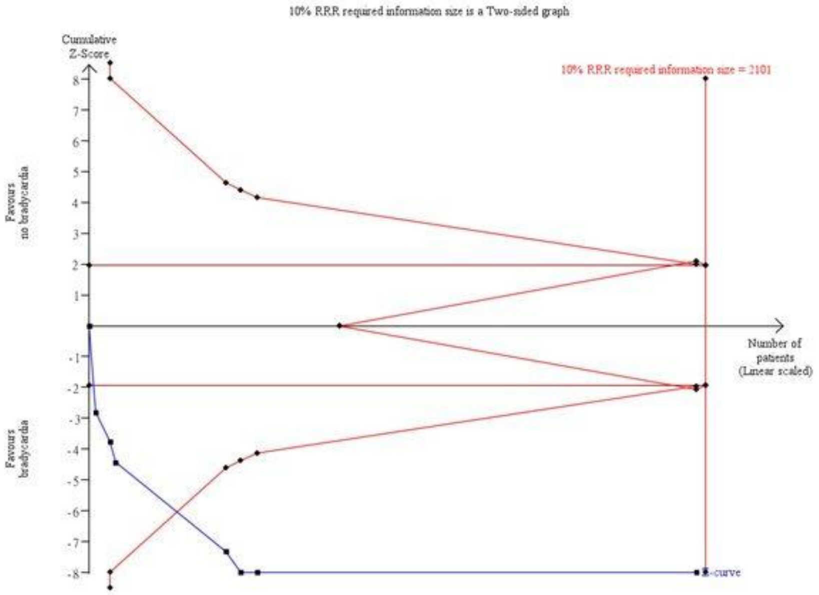

Figure S20. Trial sequential analysis of the low risk of bias in studies comparing the impact on remdesivir-induced bradycardia

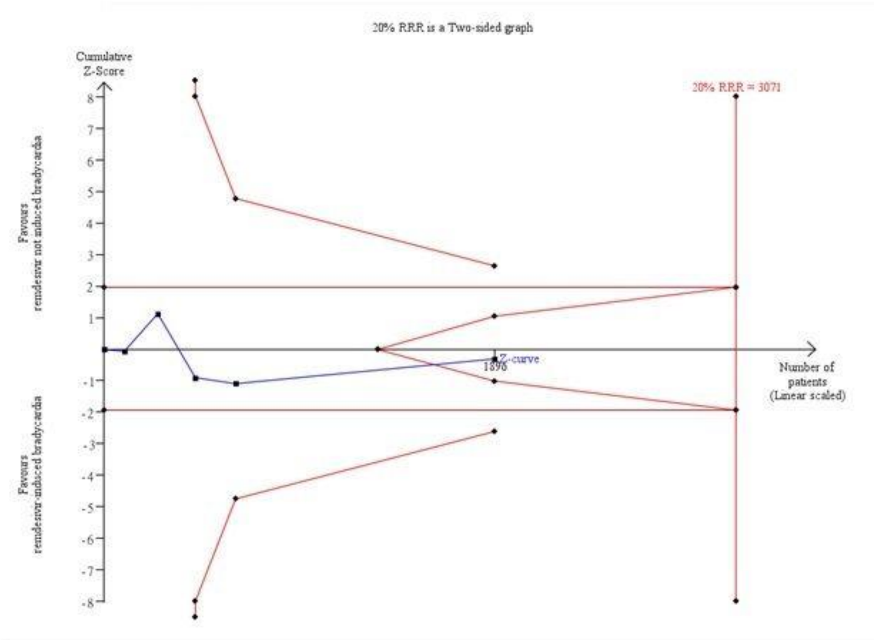

Figure S21. Trial sequential analysis of the low risk of bias in studies comparing the impact on remdesivir-induced bradycardia-related mortality.
